# Adjusting the length of supramolecular polymer bottlebrushes by top-down approaches

**DOI:** 10.3762/bjoc.17.175

**Published:** 2021-10-21

**Authors:** Tobias Klein, Franka V Gruschwitz, Maren T Kuchenbrod, Ivo Nischang, Stephanie Hoeppener, Johannes C Brendel

**Affiliations:** 1 Laboratory of Organic and Macromolecular Chemistry (IOMC), Friedrich Schiller University Jena, Humboldtstraße 10, 07743 Jena, Germany; 2 Jena Center for Soft Matter (JCSM), Friedrich Schiller University Jena, Philosophenweg 7, 07743 Jena, Germany

**Keywords:** distribution, dual centrifugation, filomicelles, self-assembly, ultrasonication

## Abstract

Controlling the length of one-dimensional (1D) polymer nanostructures remains a key challenge on the way toward the applications of these structures. Here, we demonstrate that top-down processing facilitates a straightforward adjustment of the length of polyethylene oxide (PEO)-based supramolecular polymer bottlebrushes (SPBs) in aqueous solutions. These cylindrical structures self-assemble via directional hydrogen bonds formed by benzenetrisurea (BTU) or benzenetrispeptide (BTP) motifs located within the hydrophobic core of the fiber. A slow transition from different organic solvents to water leads first to the formation of µm-long fibers, which can subsequently be fragmented by ultrasonication or dual asymmetric centrifugation. The latter allows for a better adjustment of applied shear stresses, and thus enables access to differently sized fragments depending on time and rotation rate. Extended sonication and scission analysis further allowed an estimation of tensile strengths of around 16 MPa for both the BTU and BTP systems. In combination with the high kinetic stability of these SPBs, the applied top-down methods represent an easily implementable technique toward 1D polymer nanostructures with an adjustable length in the range of interest for perspective biomedical applications.

## Introduction

Cylindrical polymer nanostructures in solution have received increasing attention during the last two decades, related to the high surface-to-volume ratio, which is particularly attractive for targeted carrier materials in biomedical applications. In addition, cylindrical drug delivery vehicles appear to be advantageous compared to the spherical analogues with regard to blood circulation time, drug loading, and tumor penetration abilities [1–3]. However, the straightforward preparation of cylindrical polymer aggregates with defined and reproducible length still remains challenging but represents a prerequisite for the desired applications in nanomedicine [4–5]. A key factor in this regard are formulation strategies, which allow a straightforward implementation into established processes that are, e.g., in accordance with good manufacturing practice (GMP) [6]. Approaches such as the crystallization-driven self-assembly (CDSA) or the synthesis of covalently bound cylindrical polymer brushes (CPBs) offer access to a defined fiber length [7–16]. However, they also suffer from disadvantages, such as significant experimental effort to evaluate suitable reaction procedures for the synthesis or the conditions for the assembly process, and are therefore often limited to specific materials. An alternative is the use of molecular motifs capable of forming directional supramolecular interaction forces, such as hydrogen bonds or π-interactions, to guide the one-dimensional (1D) assembly of established, commercial polymers in solution [17]. We recently reported the self-assembly of polyethylene oxide (PEO) polymers into cylindrical nanostructures, also called supramolecular polymer bottlebrushes (SPBs), based on the end group modification with hydrogen bond forming benzenetrisurea (BTU) and benzenetrispeptide (BTP) motifs [18–20]. The resulting amphiphilic character of the materials facilitated a control of the kinetic assembly, which provided access to stable nanostructures on a broad length range (<100 nm–2 µm). While the process enables a good adjustment of the length, it relies on a precise control of the assembly pathway and requires the use of organic solvents, such as THF and DMF, which limits the applicability in pharmaceutical formulations [21]. For an application in nanomedicine, a length in the range of 100–200 nm is particularly attractive to ensure cellular uptake and to access the known size window for a potential enhanced permeability and retention effect (EPR) [22–23]. As an alternative for the assembly pathway control, we opted to apply easy-to-use top-down approaches to tune the length distribution in a straightforward fashion over the above-mentioned length range of interest. While ultrasonication (US) represents a standard but rather harsh fragmentation technique, we additionally introduced dual asymmetric centrifugation (DAC) as an excellent alternative top-down method for effective, more controlled, and adaptable preparation of polymer nanostructures [24–28]. Both methods are applied for fragmentation of initially µm-long SPBs based on BTU–PEO and BTP–PEO conjugates. The resulting nanofibers were characterized in detail by cryogenic transmission electron microscopy (cryoTEM), as well as by asymmetrical flow field-flow fractionation measurements coupled to a UV detector and a multiangle laser light scattering detector (AF4–MALLS technique) to estimate the apparent structure length and the length distribution of the SPBs.

## Results and Discussion

**Figure 1:**
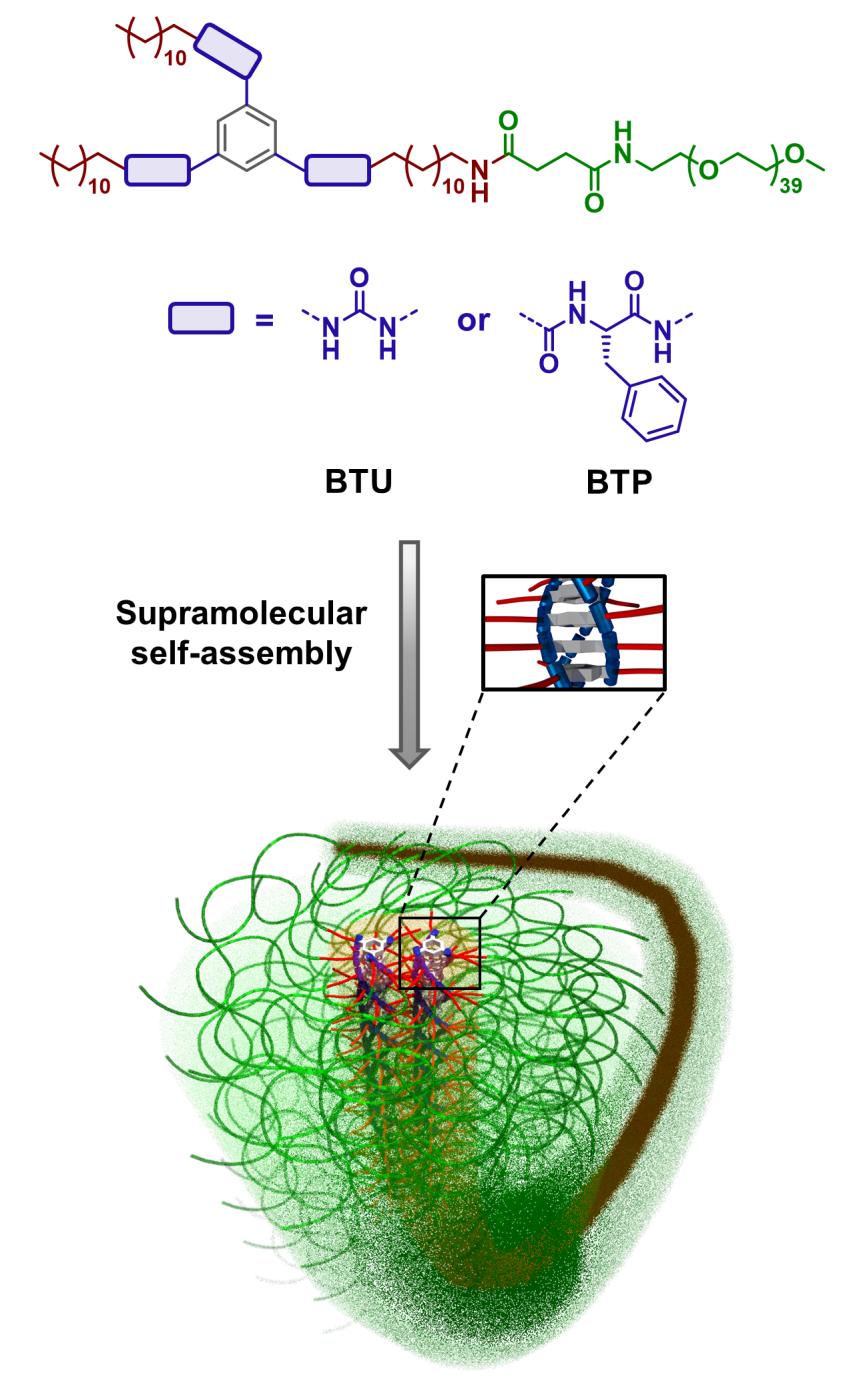
Schematic representation of the chemical structures of BTU and BTP and the supramolecular self-assembly of the compounds into SPBs.

The general structure of the tested BTP and BTU is depicted in Figure 1 [19–20]. While the hydrogen bonding moieties are either urea-based or peptide-based (i.e., phenylalanine) units, the dodecyl chains act as hydrophobic shields to induce the amphiphilic assembly in water and prevent the surrounding water from interfering with the hydrogen bonds in the interior [29]. Attaching a hydrophilic PEO chain (2 kg⋅mol^−1^), the compounds self-assemble into long fiber structures consisting of 2–4 lateral core units upon transfer into water, as reported previously [19–21]. A slow solvent change from a THF solution to water resulted in µm-long fibers in both cases (BTU or BTP).

An initial attempt to tune the fiber length by changing the initial organic solvent was not successful. As seen in the corresponding cryoTEM images (Figures S1–S6, Supporting Information File 1) as well as AF4 measurements (Figure S10, Supporting Information File 1), all tested organic solvents yielded similarly µm-long fibers, exemplifying the surprisingly minor influence of the initial organic solvent on the resulting fiber length. Alternatively, a targeted size below 1 µm can be achieved by top-down strategies inducing strong shear forces. Typically, US is applied to fragment supramolecular structures [7,30–33]. However, US causes cavitation within the sample, the collapse of which is accompanied by very high local heating. Alternative approaches rely on inducing strong shear forces by strong mixers or dispersers. An interesting method in this regard is the use of DAC, which is also considered to be a speed-mix technology due to the rapid mixing of the sample [28]. In DAC, the sample holder performs an additional rotation besides the main rotor rotation, resulting in a continuous change of the direction of the centrifugal force [28]. This change induces a strong agitation of the solution and generates large shear forces. DAC has mainly been used to create drug composites but recently found application in the formulation of liposomes or the direct nanodispersion of pharmaceutically active ingredients [24–28]. The technique resembles nanomilling methods but allows a much smaller sample scale, which renders it particularly attractive for testing the suitability to fragment fiber-like supramolecular assemblies in solution [34].

**Figure 2:**
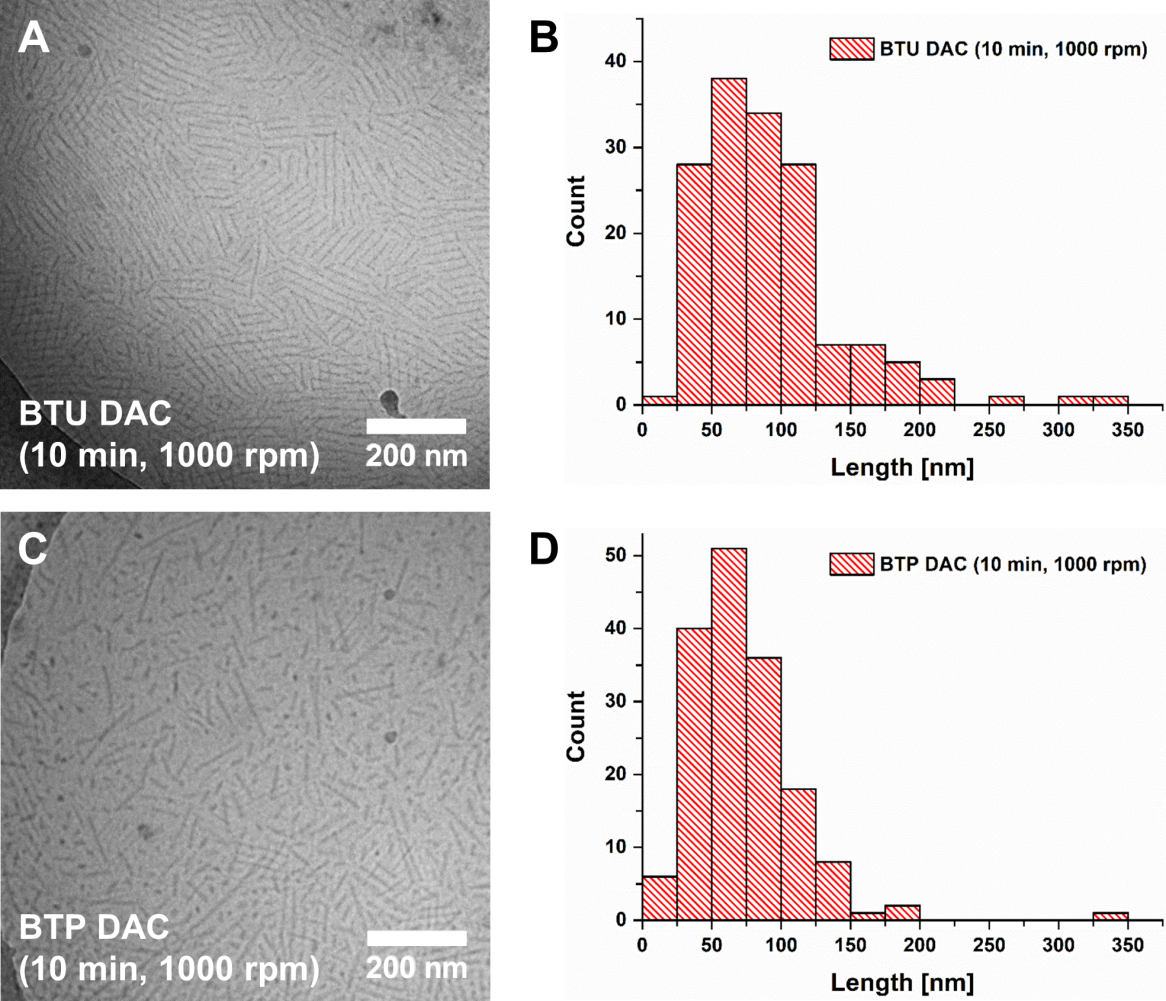
cryoTEM images of A) BTU DAC (10 min, 1,000 rpm) and C) BTP DAC (10 min, 1,000 rpm). The corresponding histograms B) and D) show the length distribution of BTU DAC (10 min, 1,000 rpm, B, mean length: 92 ± 51 nm) and BTP DAC (10 min, 1,000 rpm, D, mean length: 74 ± 39 nm).

We started with a rotational speed of 1,000 rpm and treated the initial fibers for 10 min using samples BTU DAC (10 min, 1,000 rpm) and BTP DAC (10 min, 1,000 rpm). This comparably mild treatment already caused a significant fragmentation of the µm-long fibers, resulting in structures of 50–200 nm length according to the cryoTEM images after 10 min of treatment (Figure 2). An average fiber length of 92 ± 51 nm and 74 ± 39 nm for BTU DAC (10 min, 1,000 rpm) and BTP DAC (10 min, 1,000 rpm), respectively, were apparent according to cryoTEM.

**Figure 3:**
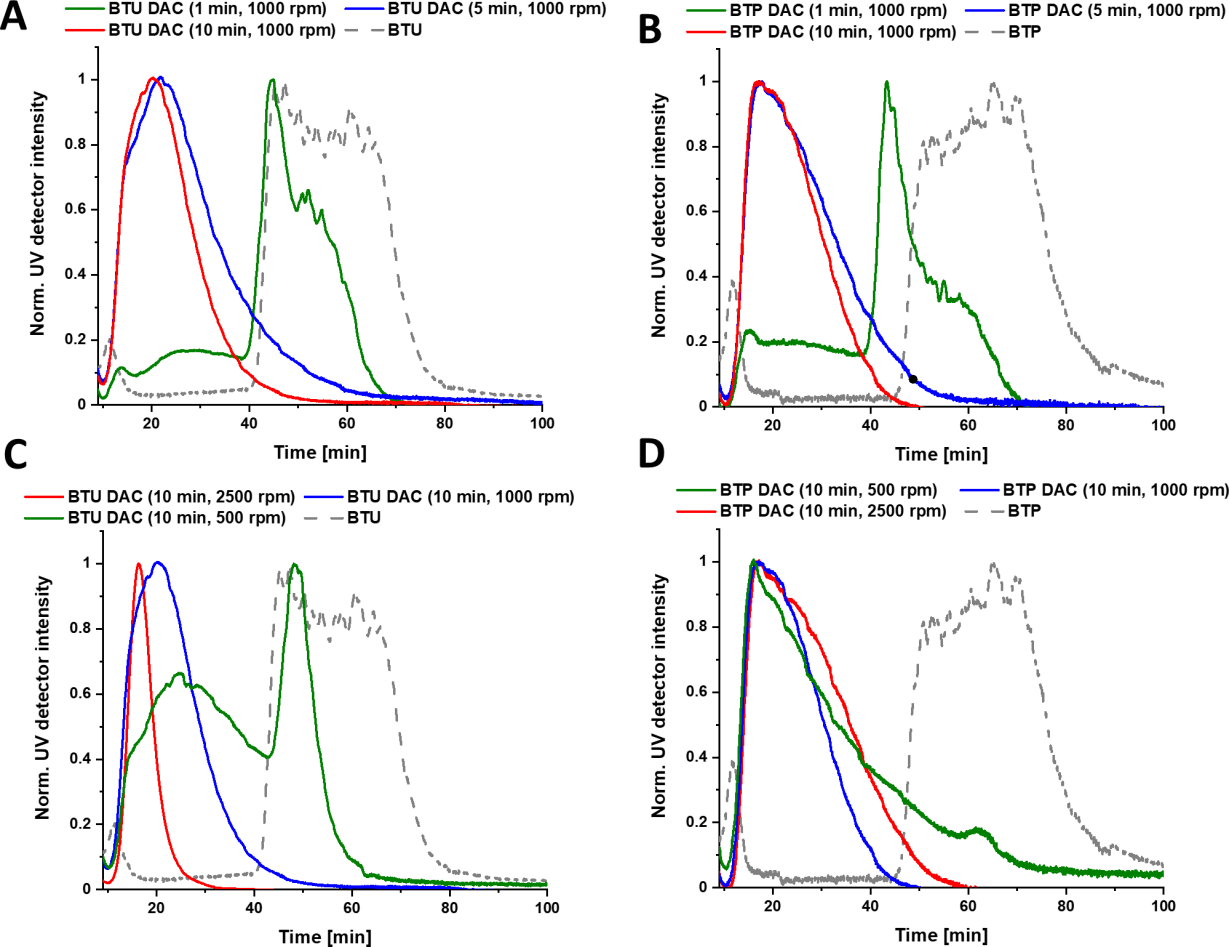
AF4 elution profiles showing the stability against dual centrifugation over different time ranges at a rotation speed of 1,000 rpm (A and B) and 2500 rpm (C and D) of BTU and BTP, respectively. Injection peaks are omitted for clarity.

This could further be supported by AF4 measurements (Figure 3A and 3B). Here, fibers featuring an average radius of gyration of *R*_g_ ≈ 20 nm and a weight-average molar mass of *M*_w_ = 3,099,000 g⋅mol^−1^, corresponding to a number of aggregation of *N*_agg_ ≈ 1,200 and a length of 110 nm for BTU (assuming four units per cross-section), as well as a weight-average molar mass of *M*_w_ = 4,406,000 g⋅mol^−1^, corresponding to a number of aggregation of *N*_agg_ ≈ 490 and a length of 120 nm (assuming two units per cross-section) for BTP, could be observed after 10 min at 1,000 rpm (Figure S12A and S12B, Supporting Information File 1) [19,21].

As a consequence, we scrutinized the influence of time and rotation speed on the size of the fragments, which were analyzed by AF4 (Figure 3). First, samples were treated for 1, 5, and 10 min at a rotation speed of 1,000 rpm (Figure 3A and 3B as well as Figure S12A and S12B, Supporting Information File 1) to investigate if an extended exposure time can break up the aggregates even further. For BTU, the peak maximum of the UV trace was shifted from 60 min to 45 min already after 1 min of mixing (Figure 3A). After 5 min of centrifugation, no further change could be observed since the peak at this low elution time already corresponded to very small oligomers (Figure 3A). For BTP, a stronger downward shift of the peak maximum from 70 to 45 min could be observed after 1 min of mixing. Similar to BTU, the increase in centrifugation time to 5 min led to a more pronounced fragmentation, which was not further enhanced by prolonged DAC.

To determine the influence of the strength of the shear forces, we also increased or decreased the centrifugal speed to 2,500 and 500 rpm, respectively (Figure 3C and 3D). At 2,500 rpm, the fragmentation of the aggregates occurred more rapidly, and a significant shift in AF4 elution time was observed for both samples, BTU and BTP, within 1 min of DAC (Figure S11A and S11B, Supporting Information File 1). For BTU, the samples were further fragmented with extended time at this speed, and the smallest structures were obtained for BTU with *R*_g_ < 3 nm (Figure S12C, Supporting Information File 1). On the contrary, the reduction of the applied shear forces (500 rpm) limited the fragmentation rate for both samples. For BTU and after 10 min, a significant amount of the large structures (>40 min elution time) remained intact (Figure 3C and 3D as well as Figure S11C and S11D, Supporting Information File 1). Overall, DAC represents a straightforward technique to adjust the size of these supramolecular assemblies, which could easily be tuned by variation of rotational speed and treatment time. Nevertheless, the distribution of the aggregate size remained rather broad. Increasing the time of treatment, the length of the fibers appeared to approach a lower size limit depending on the speed of rotation, which became particularly apparent for the BTU compounds. Even an extended mixing time of 3 h at 2,500 rpm did not significantly change the observed distributions compared to 10 min treatment (Figure S13, Supporting Information File 1).

**Figure 4:**
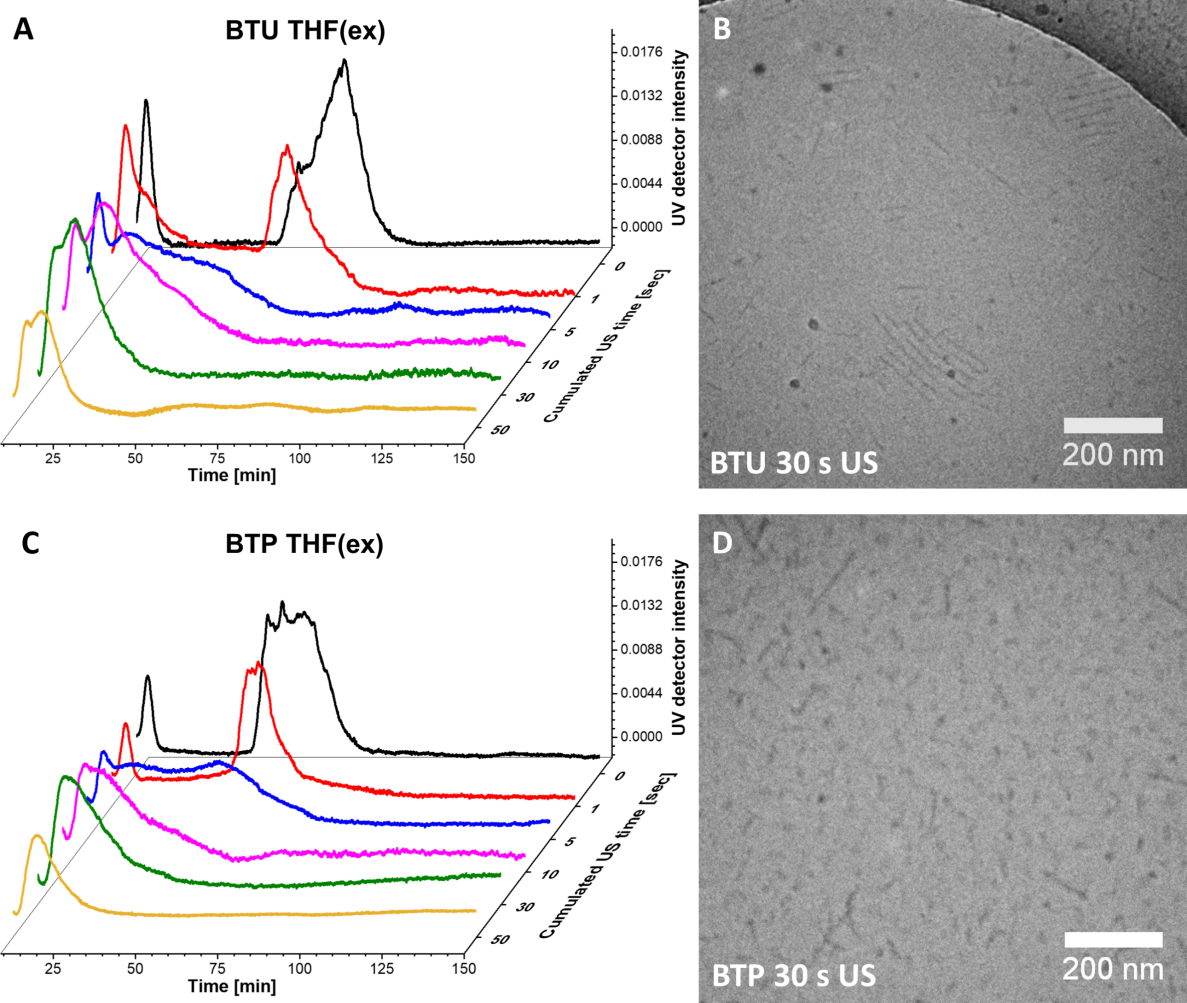
AF4−UV elution profiles after US for the cumulated time of 0 s (black), 1 s (red), 5 s (blue), 10 s (magenta), 30 s (green), and 50 s (yellow) for BTU (A) and BTP (C). The injection peaks were omitted for clarity. cryoTEM images of BTU (B) and BTP (D) after 30 s of US exposure (*c* = 1 mg⋅mL^−1^).

For comparison to more established techniques, we tested the impact of US on the same fibers. Initial tests on treating fibers within an ultrasound bath (≈11.5 W⋅L^−1^) did not reveal any changes in the structure. Therefore, we decided to apply a US probe at higher power (200 W) and varied the exposure times (Figure 4).

Already after 1 s of US, a significant decrease in the length of the fibers could be seen for both samples (Figure 4A and 4C), substantiating the rather harsh forces induced by this technique at the applied conditions. A reduction of the applied power might represent a suitable way to further limit the shear forces on the sample and gain more control, but this was not further tested. The peak maxima at around 80 min (BTU) and 65 min (BTP) decreased slightly and shifted to a lower elution time. For the BTU sample, even a new peak was formed at 10 to 20 min, corresponding to the formation of short structures. Continuous US for a cumulated time of 5 s resulted in the disappearance of the main peaks and the appearance of a broad distribution ranging from 15 min to 70 min elution time for both samples. The severe broadening of the distribution and the immediate formation of very small structures suggests a shearing off of small fragments during US. A further increase of the time (up to 30 s) narrowed the length distribution once again, and only the small aggregates remained in solution, which appeared to be stable during further sonication (50 s of cumulated US time). The resulting fibers featured an average *R*_g_ value of ≈15 nm (Figure S14, Supporting Information File 1) and a weight-average molar mass of *M*_w_ = 3,633,000 g⋅mol^−1^, corresponding to a number of aggregation of *N*_agg_ = 1400 and a length of 125 nm for BTU (assuming four units per cross-section). For BTP, a weight-average molar mass of *M*_w_ = 2,910,000 g⋅mol^−1^, corresponding to a number of aggregation of *N*_agg_ = 440 and a length of 80 nm (assuming two units per cross-section) was calculated [19,21]. Correlating well with the AF4 results, cryoTEM images of both samples (Figure 4B and 1D) showed mainly short cylinders after a cumulated US time of 30 s, with an average fiber length of 124 ± 65 nm and 69 ± 41 nm for BTU and BTP, respectively (Figure S8, Supporting Information File 1). It is important to note that all obtained fibers remained unchanged over several months after the top-down processing, demonstrating the previously described excellent kinetic stability of these supramolecular aggregates (Figures S15 and S16, Supporting Information File 1) [21].

Inspired by work of Lamour et al., we estimated a similar limit length *L*_lim_ upon extensive US treatment [35]. This length allows an indirect estimation of the tensile strength σ* of our fibers according to


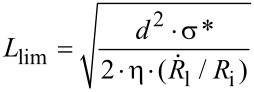


where 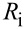 is the radius of the cavitation bubble, 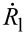 is the wall velocity of the collapsing bubble, *d* is the fiber diameter, and *η* is the viscosity of the solvent [36]. By assuming typical values for the wall velocity, bubble radius, and viscosity of the solvent, the equation simplifies [37–38] to


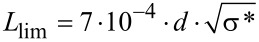


We exposed the BTU and BTP fibers to extended US (>1 h). No further scission could be observed after 2–3 h of US. AF4–MALLS measurements of the fibers after 3 h US revealed a *M*_w_ at the elution peak maximum of 843,000 g⋅mol^−1^ and 943,000 g⋅mol^−1^ for BTU and BTP, respectively (Figure S17, Supporting Information File 1). This translates to a fiber length of 29 nm for BTU by assuming a stacking distance of 0.36 nm and 4 molecules per cross-section [19], and 28 nm for BTP by assuming a similar stacking distance and 2 molecules per cross-section [21]. Based on a fiber diameter of 12 nm (estimated from small-angle X-ray scattering experiments) [19–20], the resulting tensile strength for both compounds was approximately 16 to 17 MPa (Table S1, Supporting Information File 1). This strength was in the range of Elastin filaments and significantly lower as, for instance, the tensile strength of amyloid fibrils [35,39]. Overall, the observed values for the fibers corresponded well to the sensitivity to shear forces. However, the core–shell structure of our supramolecular systems has to be considered in this regard. For example, significant steric strains induced by the polymer chains act on the core structure, limiting the strength of the supramolecular assembly.

## Conclusion

In conclusion, the remarkable long-term stability of BTU–PEO and BTP–PEO fibers in water, which were prepared via bottom-up self-assembly, enabled us to apply two straightforward top-down approaches (US and dual asymmetric centrifugation) to tune the length distributions of the supramolecular fibers. Exposing the SPBs to US resulted in a rapid fragmentation of the fibers into small rod-like fragments. Dual asymmetric centrifugation, on the other hand, allowed to adjust the length distribution in a more controlled manner by adjusting the time and rotation speed. Thus, this study demonstrates that easy-to-use top-down methods can be a feasible approach to obtain some control over the length distributions of 1D polymer nanostructures, and thus this makes them more likely to be applied in biomedicine, where dimensional control is a prerequisite.

## Supporting Information

File 1 Synthesis, procedures, and characterization.
