# Supplementary material for: Adjusting the length of supramolecular polymer bottlebrushes by top-down approaches
Source: Beilstein J Org Chem. 2021 Oct 21;17:2621–8. doi: 10.3762/bjoc.17.175 (PMC8551873; doi:10.3762/bjoc.17.175)
Supplement: File 1 — Synthesis, procedures, and characterization. [file Beilstein_J_Org_Chem-17-2621-s001.pdf]

BEILSTEIN JOURNAL OF ORGANIC CHEMISTRY

Supporting Information

for

Adjusting the length of supramolecular polymer bottlebrushes by top-down approaches

Tobias Klein, Franka V. Gruschwitz, Maren T. Kuchenbrod, Ivo Nischang, Stephanie Hoeppener and Johannes C. Brendel

Beilstein J. Org. Chem. **2021,** *17,* 2621–2628. doi:10.3762/bjoc.17.175

Synthesis, procedures, and characterization

License and Terms: This is a supporting information file under the terms of the Creative Commons Attribution License (https://creativecommons.org/licenses/by/4.0). Please note that the reuse, redistribution and reproduction in particular requires that the author(s) and source are credited and that individual graphics may be subject to special legal provisions.
The license is subject to the *Beilstein Journal of Organic Chemistry* terms and conditions: (https://www.beilstein-journals.org/bjoc/terms)

Content

1. Synthesis and procedures S2

2. Characterization S3

   2.1 Cryogenic transmission electron microscopy (cryoTEM) S3

   2.2 Asymmetrical flow field-flow fractionation (AF4) S12

3. References S19

1. Synthesis and procedures

Materials and Methods

All reagents and solvents were commercial products purchased from Sigma-Aldrich, abcr, Iris BioTech, Rapp Polymere, or TCI and were used without further purification.

Synthesis

The benzenetrisurea–polyethylene oxide (BTU) and benzenetrispeptide–polyethylene oxide (BTP) conjugates were synthesized according to previously published protocols [1].

Self-assembly procedures

Solvent switch method

The self-assembly was conducted according to previous publications [2], where 5 mg of BTU or BTP were dissolved in 1 mL of THF and stirred overnight to guarantee complete dissolution. To this, 4 mL of MilliQ water were added (1–100 $mL \cdot h^{-1}$) using a syringe pump under vigorous stirring to reach a final water content of 80 vol %. For this purpose, the needle of the syringe was immersed in the organic BTP or BTU solution to enable a constant release of MilliQ water from the syringe and to avoid the formation of drops that would result in high local water concentrations at the spot where the drop immerses into the solution. Afterwards, the solution was transferred to float-a-lyzer® tubings with a molecular weight cutoff of 3.5 kD and dialyzed for five days against water to remove all THF traces.

Ultrasonication procedure

BTP and BTU samples assembled from THF at a concentration of 1 $mg \cdot mL^{-1}$ in MilliQ water were exposed to ultrasonication using a sonication probe (Hielscher ultrasonic processor UP200St, 100% amplitude, 20 W) for different durations up to a cumulated time of 50 s (1 s + 2 s + 2 s + 5 s + 5 s + 15 s+ 20 s).

For the experiments concerning the mechanical stability, the maximal power of 200 W of the ultrasonicator was applied for periods of 5 s, with a 3 s pause for 59 min each time.

Dual centrifugation procedure

Dual centrifugation was performed using a Hettich ZentriMix 380 R equipped with a ZentriMix rotor. BTU and BTP at a concentration of 1 mg·mL^{-1} in MilliQ water were added to 2 mL vials and centrifuged for different durations (1 min, 5 min, 10 min, and 180 min) at different rotation speeds (500 rpm, 1,000 rpm, and 2,500 rpm). No milling beads were added to the vials, to rely just on the shear forces caused by the dual rotor setup.

2. Characterization

2.1 Cryogenic transmission electron microscopy (cryoTEM)

Samples were prepared on Ar plasma treated Quantifoil grids (R2/2). 8.5 µL of the solutions (3 mg·mL^{-1} in H_2O) were applied onto the grids and vitrified in liquid ethane utilizing a FEI Vitrobot Mark IV system (offset: −3 mm, blotting time: 1 s). Samples were transferred into the cryo holder (Gatan 626) utilizing the Gatan cryo stage, followed by transfer into the microscope keeping the temperature below −172 °C during the whole transfer and measurement process after vitrification. Measurements were performed using a FEI Technai G^2 20 operated at an acceleration voltage of 200 kV. Images were acquired with a Mega View (OSIS, Olympus Soft Imaging Systems) or an Eagle 4k CCD camera. cryoTEM images in this study display only specific regions of interest that are representative for the whole sample.

BTU acetone

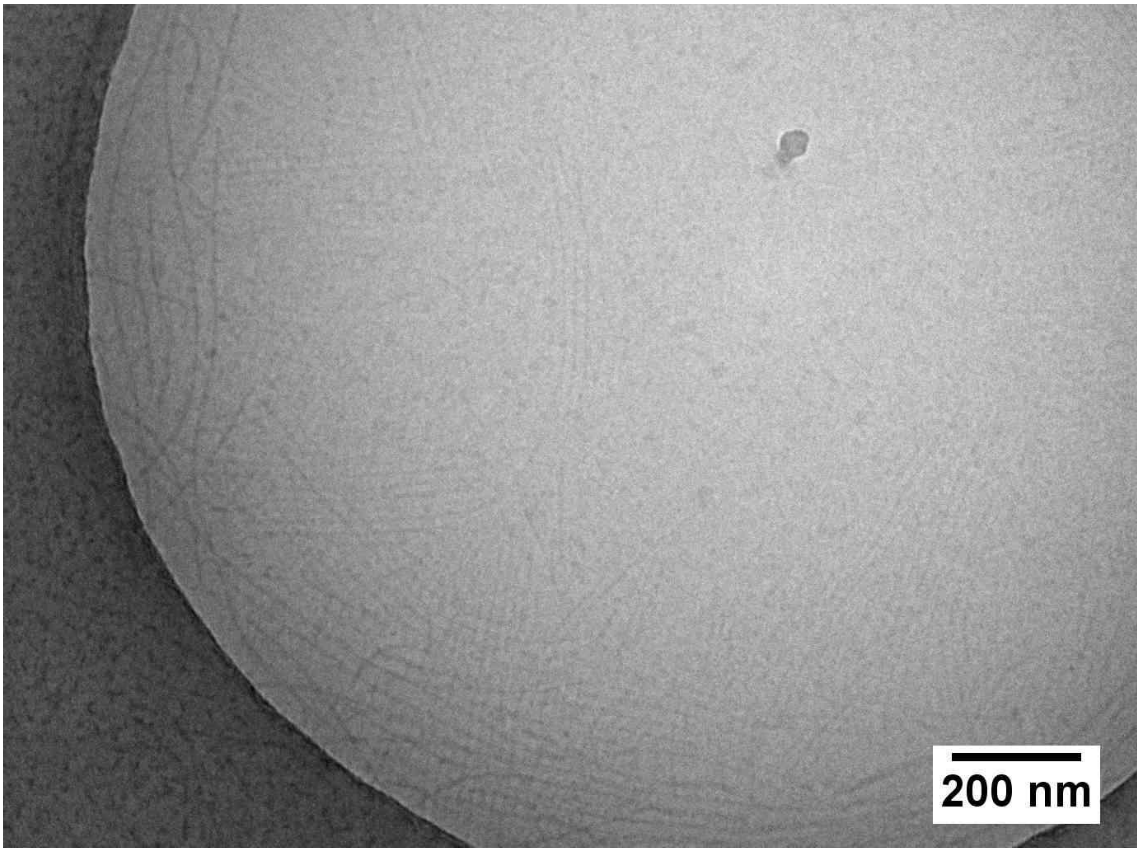

Figure S1: cryoTEM image of BTU acetone in water (c = 1 mg·mL^{-1}) assembled at 1 mL·h^{-1} water addition rate.

BTU DMF

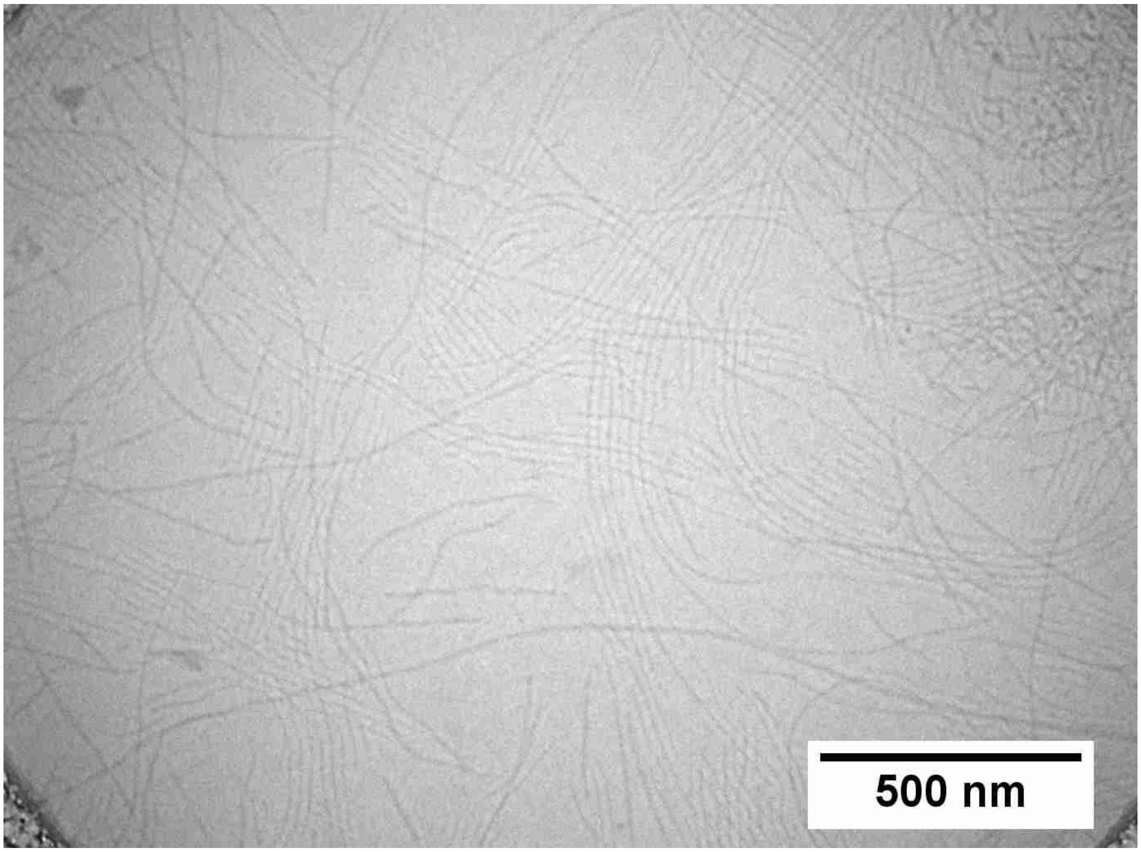

Figure S2: cryoTEM image of BTU DMF in water (c = 1 mg·mL^{-1}) assembled at 1 mL·h^{-1} water addition rate.

BTU EtOH

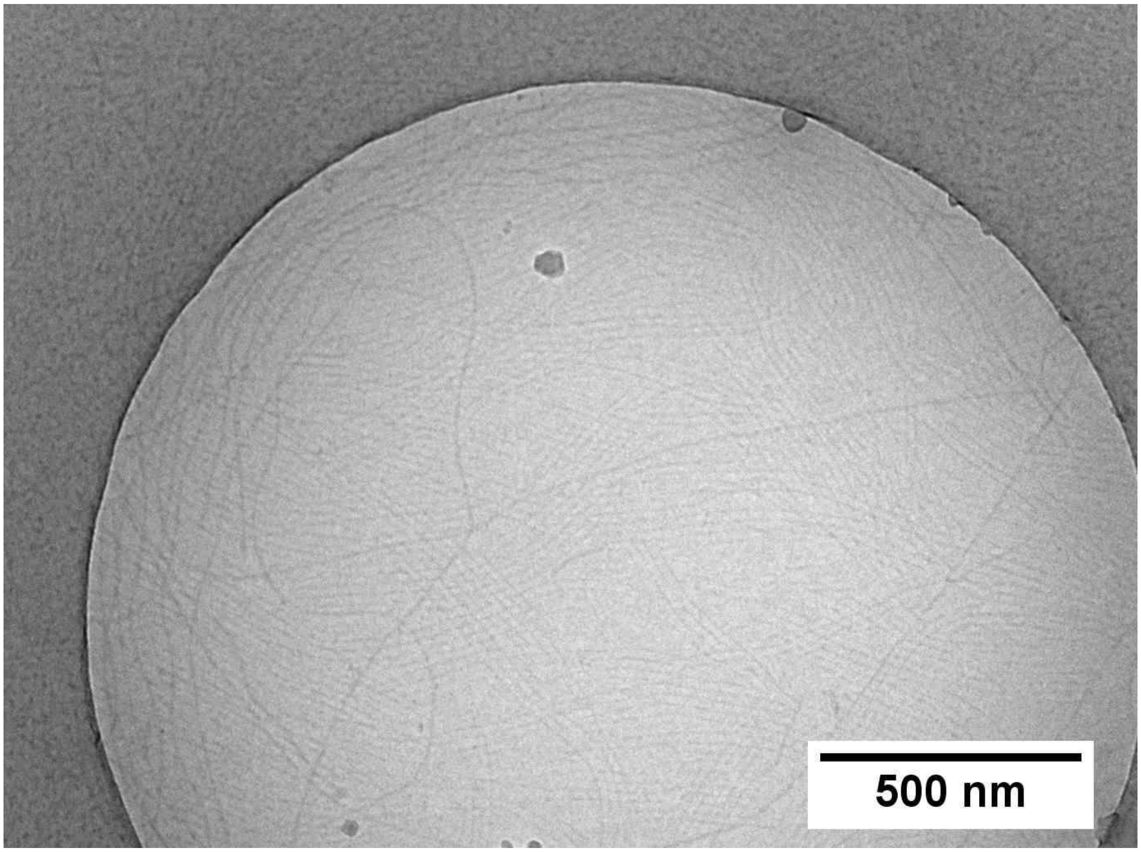

Figure S3: cryoTEM image of BTU EtOH in water (c = 1 mg·mL^{-1}) assembled at 1 mL·h^{-1} water addition rate.

BTP acetone

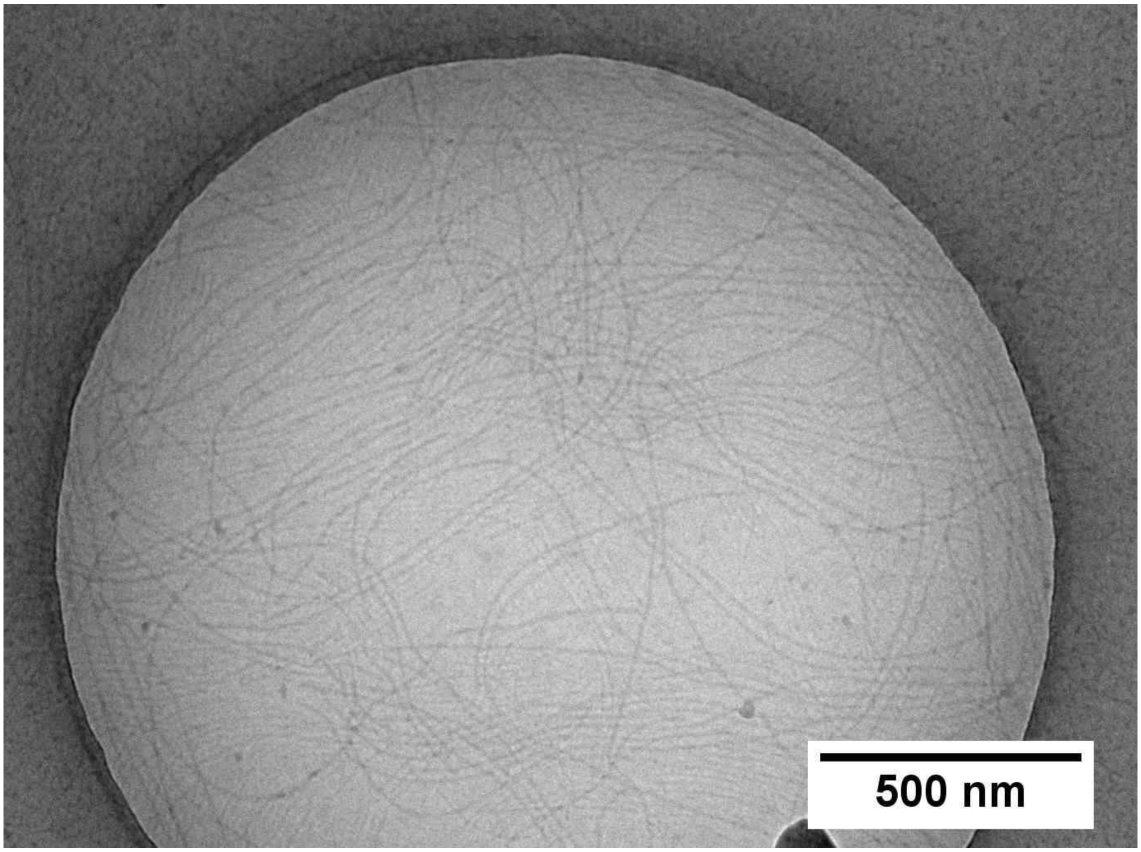

Figure S4: cryoTEM image of BTP acetone in water (c = 1 mg·mL^{-1}) assembled at 1 mL·h^{-1} water addition rate.

BTP DMF

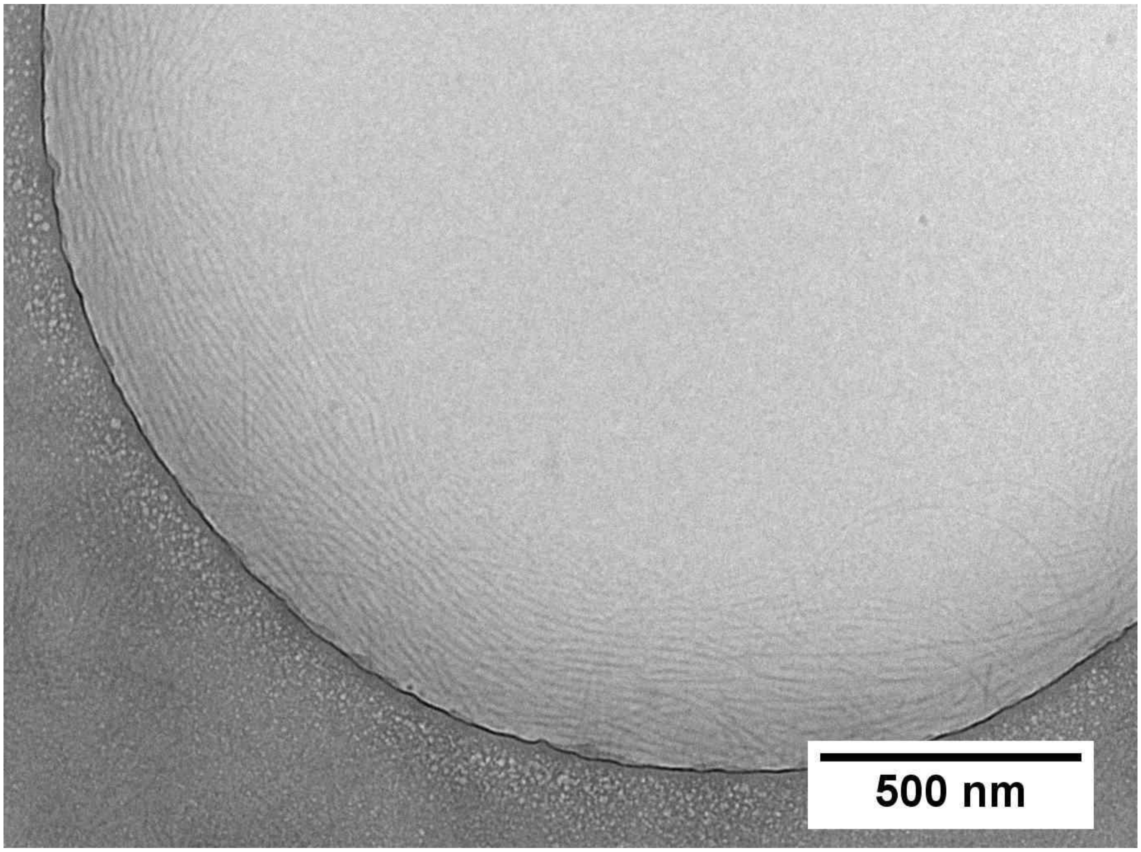

Figure S5: cryoTEM image of BTP DMF in water (c = 1 mg·mL^{-1}) assembled at 1 mL·h^{-1} water addition rate.

BTP EtOH

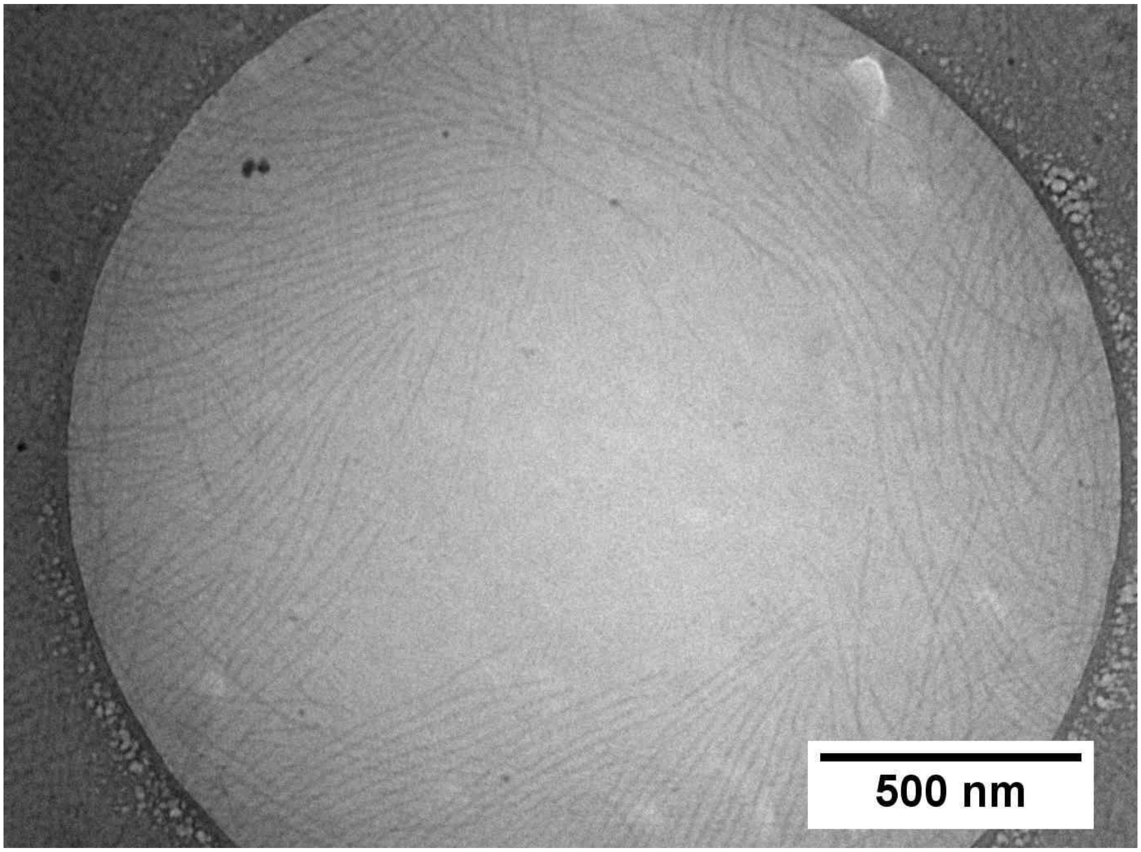

Figure S6: cryoTEM image of BTP EtOH in water (c = 1 mg·mL^{-1}) assembled at 1 mL·h^{-1} water addition rate.

BTP US 1 s

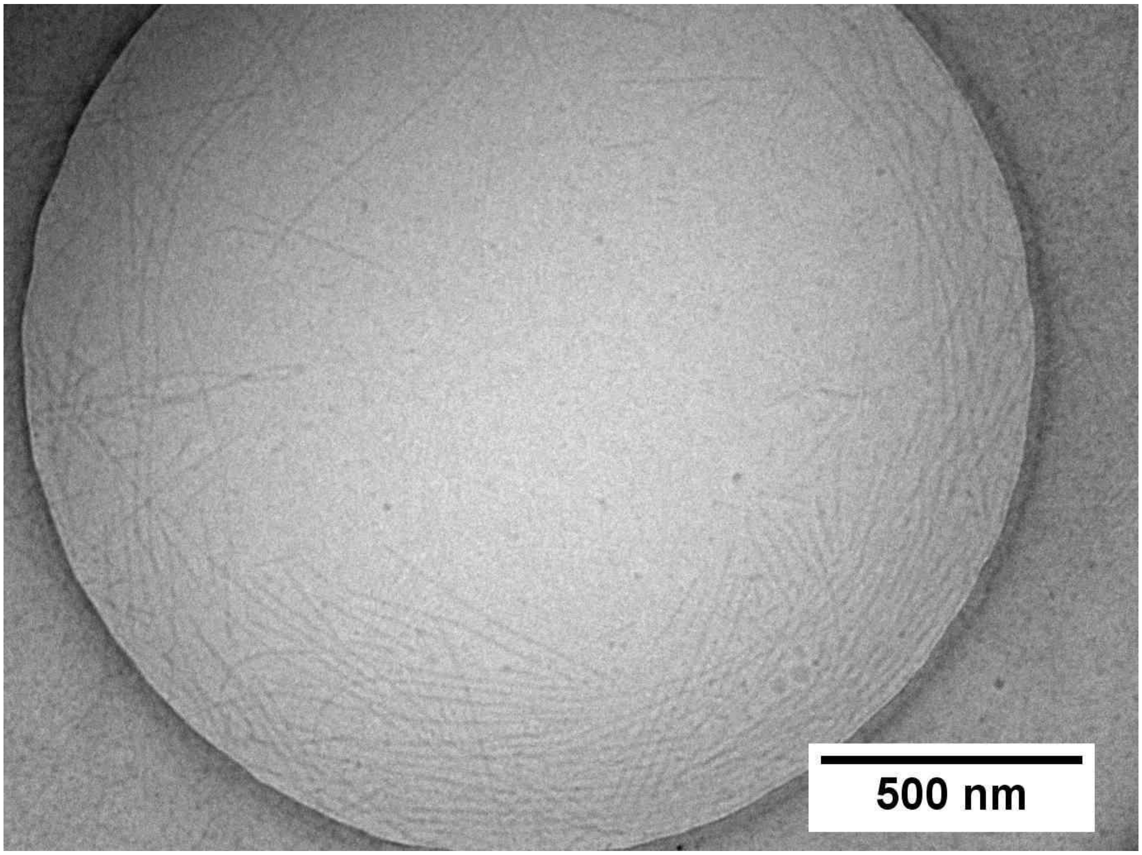

Figure S7: cryoTEM image of BTP after ultrasonication for a cumulated time of 1 s in water (*c* = 1 mg·mL^{-1}).

BTU US 30 and BTP US 30 s: cryoTEM histograms

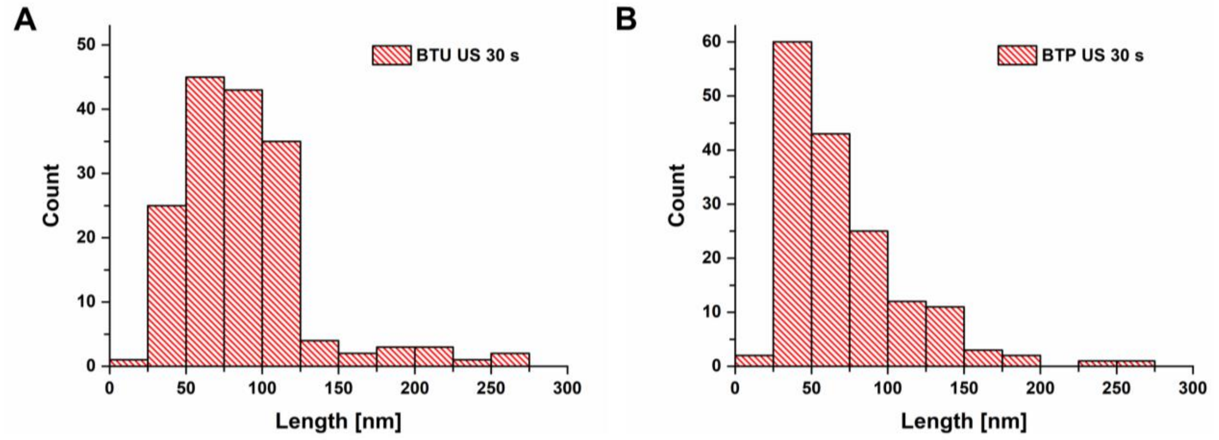

Mean length	124 ± 65 nm
Min. length	25 nm
Max. length	407 nm

Mean length	69 ± 41 nm
Min. length	15 nm
Max. length	256 nm

Figure S8: cryoTEM histograms for BTU US 30 s (A) and BTP US 30 s (B).

2.2 Asymmetrical flow field-flow fractionation (AF4)

AF4 measurements were performed on an AF2000 MT System from Postnova Analytics GmbH (Landsberg, Germany), equipped with a tip and focus pump (PN1130), an autosampler (PN5300), and a channel oven unit (PN4020) set to 25 °C. The channel was coupled to a multiangle laser light scattering (MALLS) detector (PN3621) equipped with a 532 nm laser and an overall of 21 angles (only 28°–148° have been used for calculation of R_g and M_w), a refractive index (RI) detector (PN3150), and a UV detector (PN3212) operating at a wavelength of 280 nm. The channel had a trapezoidal geometry with a nominal height of 350 µm. A regenerated cellulose (RC) membrane from Postnova Analytics GmbH (10 kDa RC membrane) with a molar mass cutoff of 10 kDa was used as the accumulation wall. As the mobile phase, an aqueous solution with 0.002 wt % of NaN_3 was used. 50 µL of the sample at a concentration of 1 mg·mL^{-1} was injected with an injection flow rate of 0.2 mL·min^{-1}, a focus flow rate of 0.8 mL·min^{-1}, and a cross-flow rate of 0.7 mL·min^{-1}, resulting in a detector flow rate of 0.3 mL·min^{-1}. The focusing time was 4 min before switching to elution at an exponentially decaying crossflow from 0.7 mL·min^{-1} to 0.2 mL·min^{-1} within 76.2 min. Thereafter, the crossflow profile was set to decay in a linear way from 0.05 mL·min^{-1} to 0.04 mL·min^{-1} within 71 min (Figure S9). Before the start of the next measurement, a rinsing step was performed at 1.5 mL·min^{-1} tip flow for 20 min. After each sample measurement, a blank measurement at identical detector conditions was run, which was subtracted from the traces of the sample measurement for analysis. The RI detector was used as the concentration-sensitive detector (dn/dc$_{BTU}$ 1.47 mL/g and dn/dc$_{BTP}$ 1.48 mL/g), and the MALLS data was analysed via a ZIMM plot analysis to obtain the R_g values and the molar mass at the specified elution times.

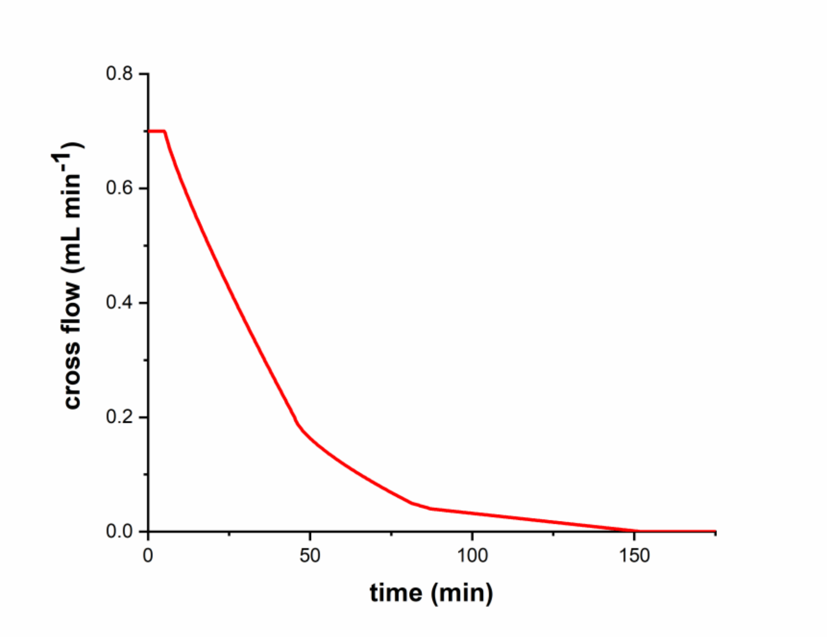

Figure S9: Cross-flow profile applied for all AF4–MALLS measurements in this manuscript.

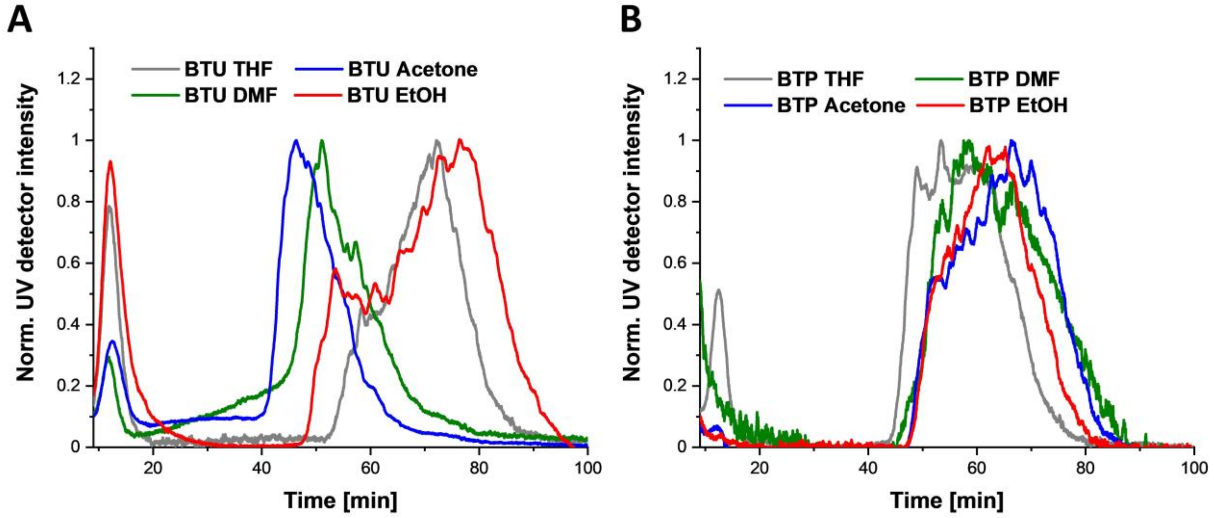

Figure S10: Normalized UV traces at 280 nm of BTU (A) and BTP (B) assembled from different organic solvents. The injection peaks were omitted for reasons of clarity.

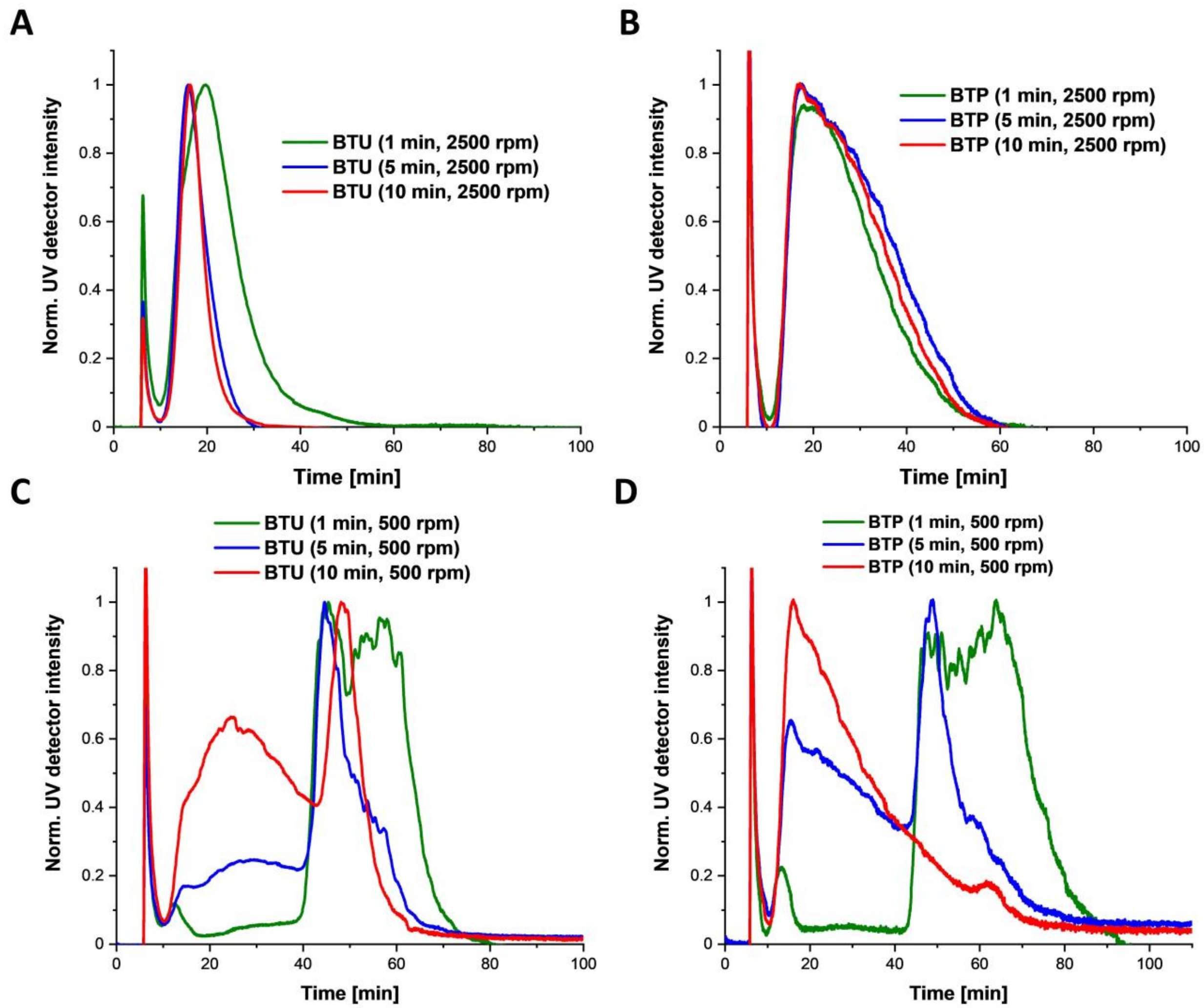

Figure S11: AF4 elution profile showing the stability against dual centrifugation at different rotation speeds of 2,500 rpm (A and B) and 500 rpm (C and D) of BTU and BTP, respectively.

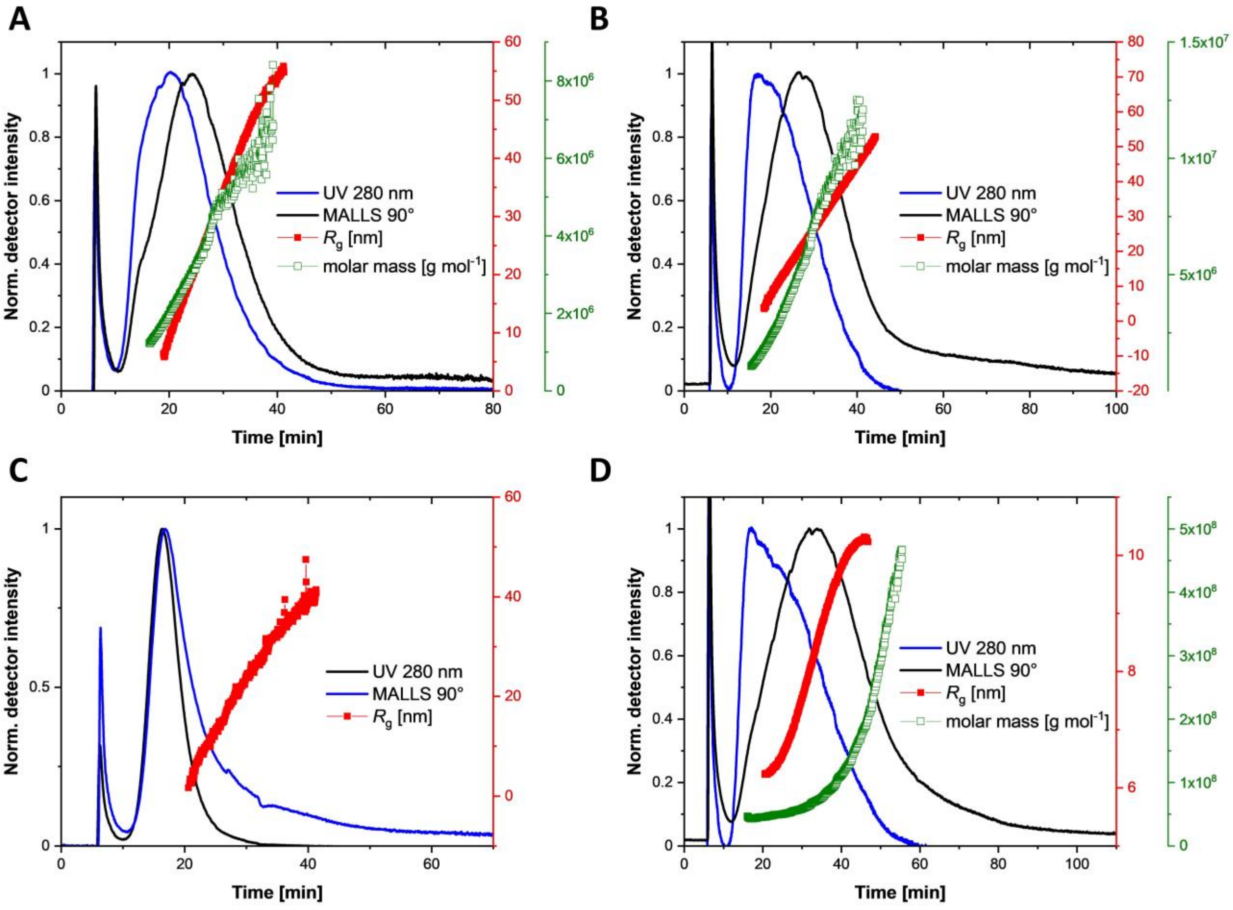

Figure S12: Normalized MALLS 90° (black) and UV (blue) traces of BTU at 1,000 rpm (A) and 2,500 rpm (C), and BTP at 1,000 rpm (B) and 2,500 rpm (D) for 10 min each. R_g (red) and molar mass (green) obtained via the Zimm plot of light scattering data from AF4–MALLS measurements. For BTU (10 min, 2,500 rpm) (C), estimation of the molar mass was not possible.

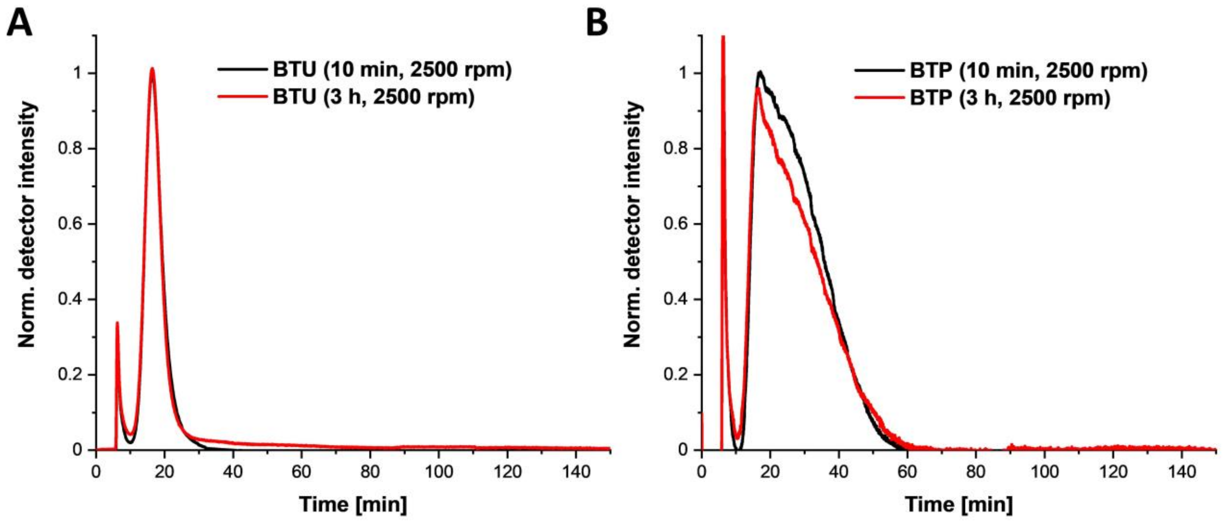

Figure S13: AF4–UV traces (at 280 nm) of BTP (A) and BTU (B) after extended centrifugation times of 3 h.

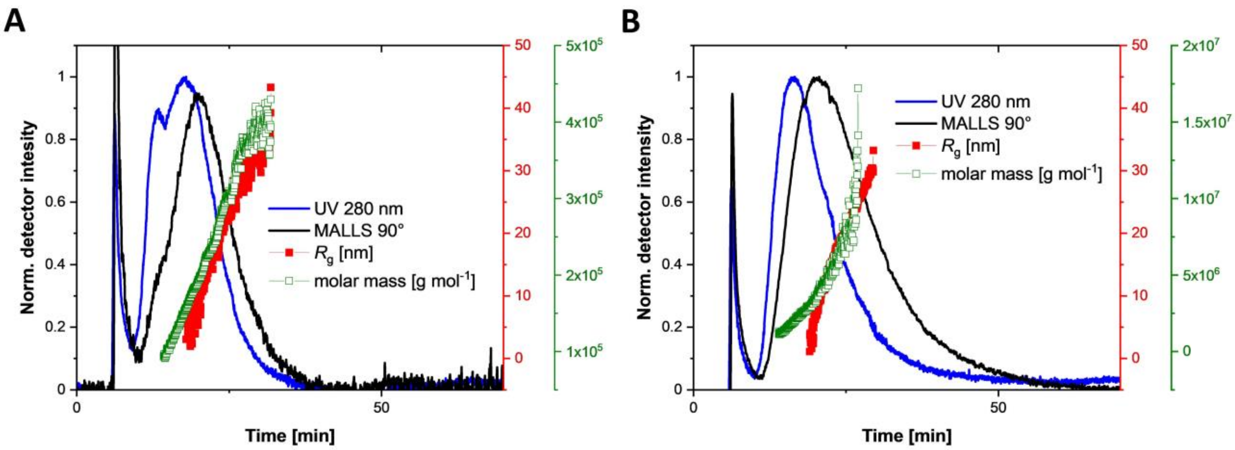

Figure S14: Normalized MALLS 90° (black) and UV (blue) traces **of BTU (A) and BTP (**B), after a cumulated time of 50 s ultrasonication (black). R_g (red) and molar mass (green) obtained via the Zimm plot of light scattering data from AF4–MALLS measurements.

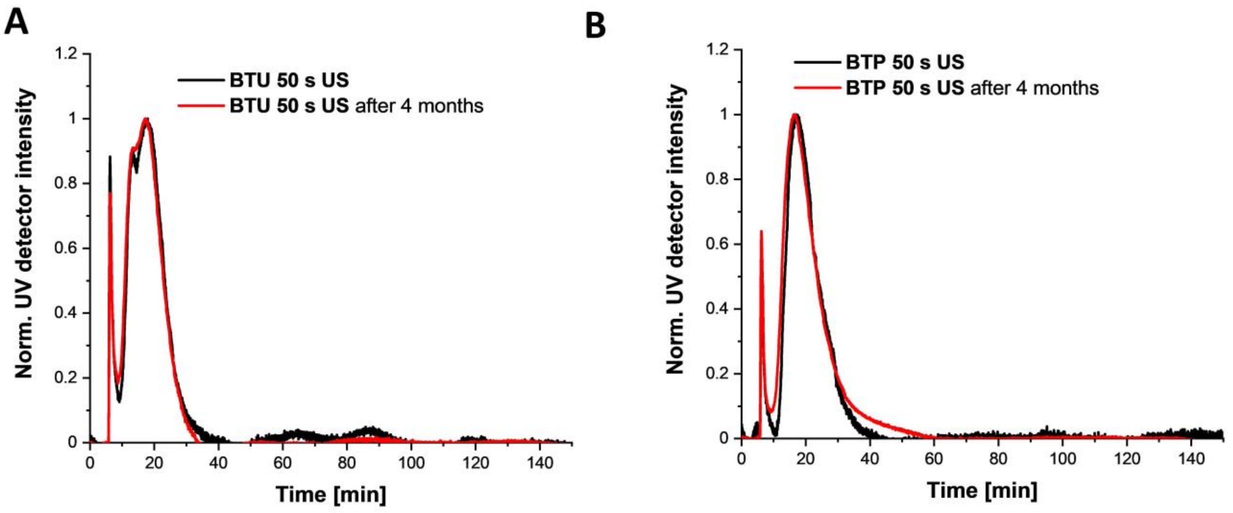

Figure S15: Stability of the samples BTP THF (A) and BTU THF (B) after 50 s of ultrasound exposure measured directly afterwards (black) and after four months (red).

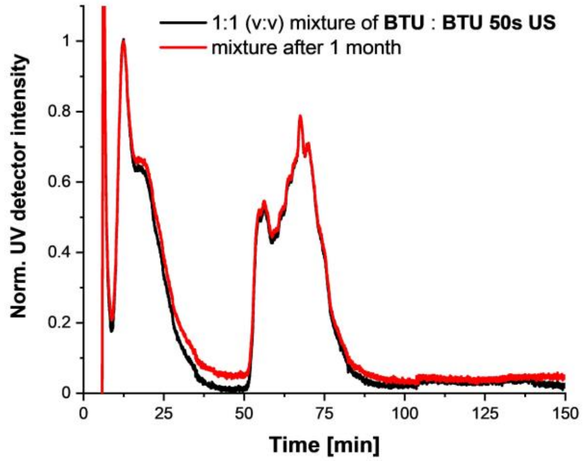

Figure S16: AF4 elution profile proving the stability of a 1:1 (v/v) mixture of BTU 50 s US and BTU over months. Slight deviations in the traces might result from variations in the membrane and pressure differences between the measurements in the AF4 setup.

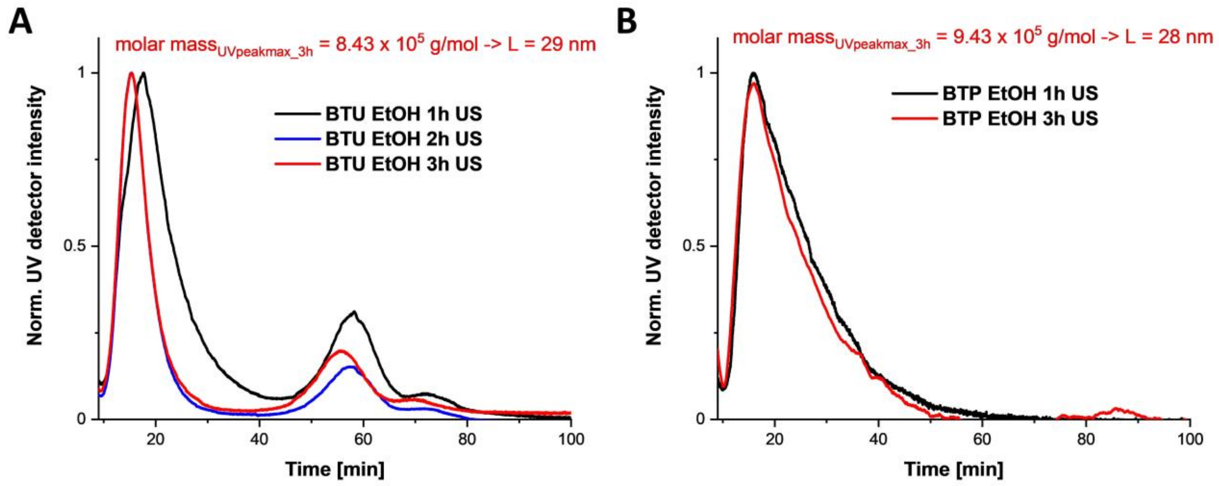

Figure S17: AF4–UV traces of BTU (A) and BTP (B) after extended ultrasonication exposures of 1 to 3 h. The injection peaks were omitted for clarity. The molar mass at the UV peak maximum (280 nm) of the elution curve after 3 h was calculated by Zimm plotting the data obtained by the MALLS detector. The length was estimated from the number of aggregation and the literature known stacking distance of the unimers of approximately 0.36 nm. After correction by the number of molecules in the cross-section, as published recently [2], an approximate length of the assemblies at this point of elution can be calculated.

Calculation of σ* from the obtained limit length after US

$$L_{lim} \approx 7 * \ 10^{-4}\, d \sqrt{\sigma^*} \qquad \text{Eq. 1}$$

$$\sigma^* \approx \left(\frac{L_{lim}}{7*10^{-4}*d}\right)^2 \qquad \text{Eq. 2}$$

Table S1: Values calculated for σ* for BTU and BTP from the respective limit length after 3 h of US.

	L_{lim} (nm)	σ* (MPa)
BTU	29	17
BTP	28	16

3. References

[1] a) F. V. Gruschwitz, M.-C. Fu, T. Klein, R. Takahashi, T. Higashihara, S. Hoeppener, I. Nischang, K. Sakurai, J. C. Brendel, *Macromolecules* **2020**, *53*, 7552-7560; b) T. Klein, H. F. Ulrich, F. V. Gruschwitz, M. T. Kuchenbrod, R. Takahashi, S. Fujii, S. Hoeppener, I. Nischang, K. Sakurai, J. C. Brendel, *Polym. Chem.* **2020**, *11*, 6763-6771.

[2] F. V. Gruschwitz, T. Klein, M. T. Kuchenbrod, N. Moriyama, S. Fujii, I. Nischang, S. Hoeppener, K. Sakurai, U. S. Schubert, J. C. Brendel, *ACS Macro Lett.* **2021**, 837-843.
